# Epispadic accessory urethra in an 8-month-old female with urethral duplication: a case report

**DOI:** 10.1097/MS9.0000000000002688

**Published:** 2024-10-23

**Authors:** Ayed S. Askar, Tarraf A. Altarraf, Shrouk F. Mohamed

**Affiliations:** a Idlib University Hospital, Syria; b Alexandria Faculty of Medicine, Egypt

**Keywords:** congenital anomaly, cystourethrography, epispadias, urethral duplication

## Abstract

**Introduction and importance:**

Urethral duplication is an uncommon urogenital congenital disability that presents as an accessory urethra in an atypical location. This condition is often coupled with other congenital anomalies like bladder exstrophy, pseudohermaphroditism, and renal disorders.

**Case presentation:**

The authors report a case of a female infant aged 8 months with urethral duplication, showing an unusual urinary opening in the prepubic area and urinary discharge. A comprehensive diagnostic assessment, consisting of a clinical exam, imaging tests, and cystoscopy, verified the existence of an accessory urethra and the normal one in epispadias. The patient had a successful surgery to remove the accessory tract with primary closure.

**Clinical discussion:**

Urethral duplication is associated with diverse clinical signs and symptoms. Accurate diagnosis needs thorough imaging studies, and treatment should be tailored according to the type and anatomical location of the accessory tract.

**Conclusion:**

The histological confirmation of urethral duplication was obtained following the surgical excision of the additional tract. This case underscores the significance of accurate diagnosis and treatment approaches to manage this case.

## Background

Highlights

- Female urethral duplication with an epispadias accessory urethra is a rare congenital anomaly.
- An 8-month-old female had a prepubic opening with normal vaginal and labial anatomy.
- Diagnostic imaging and cystoscopy confirmed urethral duplication with no other structural abnormalities.
- Surgical intervention involved excision of the accessory urethra and primary closure without compromising surrounding structures.
- Histopathological evaluation confirmed duplicated urethra with mild inflammation.
- Postoperative follow-up showed no complications and good wound healing.

Female epispadias accessory urethra is an extremely rare abnormality, occurring in around 1 out of every 480 000 girls^1^. Urethral duplication in females can occur with or without other anomalies such as ambiguous genitalia, double vagina, bifid clitoris, renal disorders (e.g. duplicated kidneys, horseshoe kidney, and hydronephrosis), bladder abnormalities (e.g. bladder exstrophy, bladder diverticula, vesicoureteral reflux, and bladder outlet obstruction), spinal disorders (e.g. spinal dysraphism and tethered cord syndrome), and rectal anomalies (e.g. imperforate anus and rectovaginal fistula). Understanding this congenital anomaly requires a deep knowledge of the morphological and embryological aspects of the female urethra, which has a regular course from the neck of the urinary bladder to the external urethral meatus. Urethral duplication has an embryological origin whereby the abnormality of the urethral fold during development fusion leads to conditions such as epispadias^2–4^.

Children with urethral duplication may exhibit symptoms such as urinary tract infections, urine retention, abnormal patterns of stream urination, and other associated malformations of the perineum. Diagnosis is conducted by careful clinical examination, radiological assessment using ultrasound and voiding cystourethrogram (VCUG), and endoscopic evaluation of the structures and function of the urinary system, cystoscopy^5–8^.

The management of female patients suffering from epispadias with urethral duplication is surgical and seeks appropriate outcomes, functional and cosmetic, using one-stage reconstruction. Such abnormalities include urethroplasty with bladder augmentation and opening plastic manipulation involved with the vaginal neck, labia minora, clitoris, and vagina if indicated. It is presented as one of the essential conditions for recovery of urinary incontinence and the restoration of anatomical significance^9^. Our case follows Surgical Case Report (SCARE) guidelines^10^.

## Case presentation

**Figure 1:**
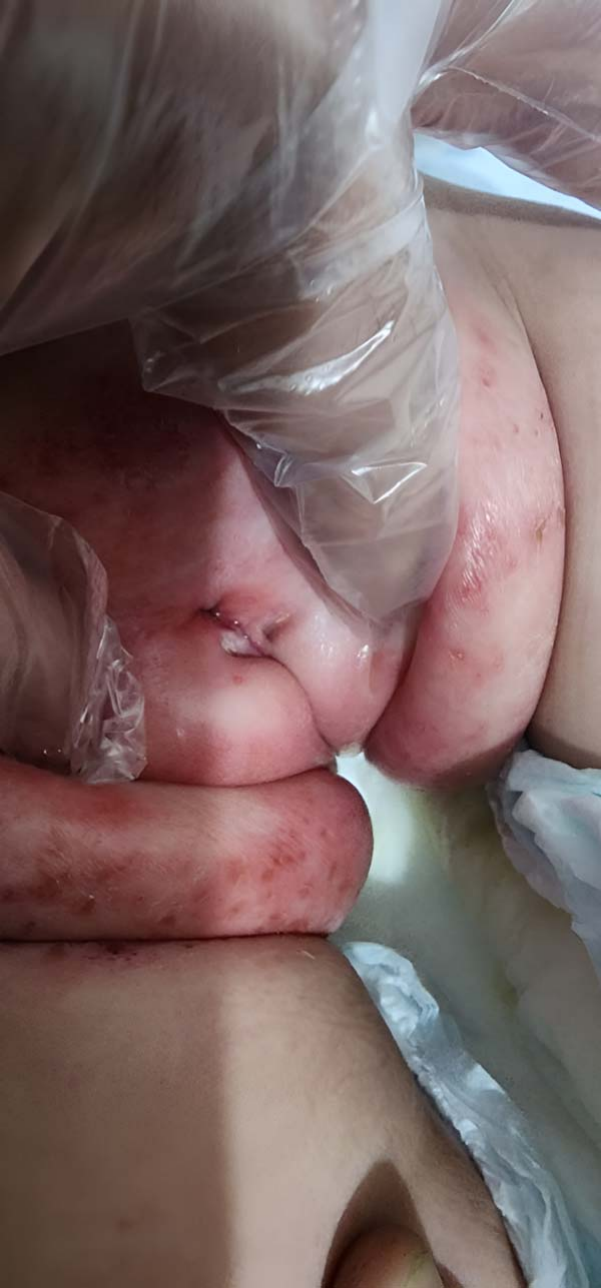
Prepubic opening and inflammation of the pubic region.

An 8-month-old female child was referred to our hospital for evaluation due to an abnormal prepubic opening noted by her parents with irritability and skin inflammation in the pubic region. On physical examination, normal vaginal and labial anatomy was observed, along with a urethral opening situated below the clitoris. The symphysis pubis remained intact and did not experience any separation. Additionally, there was visible skin irritation, and no associated genitourinary abnormalities were evident during the initial assessment, as shown in Figure 1.

**Figure 2:**
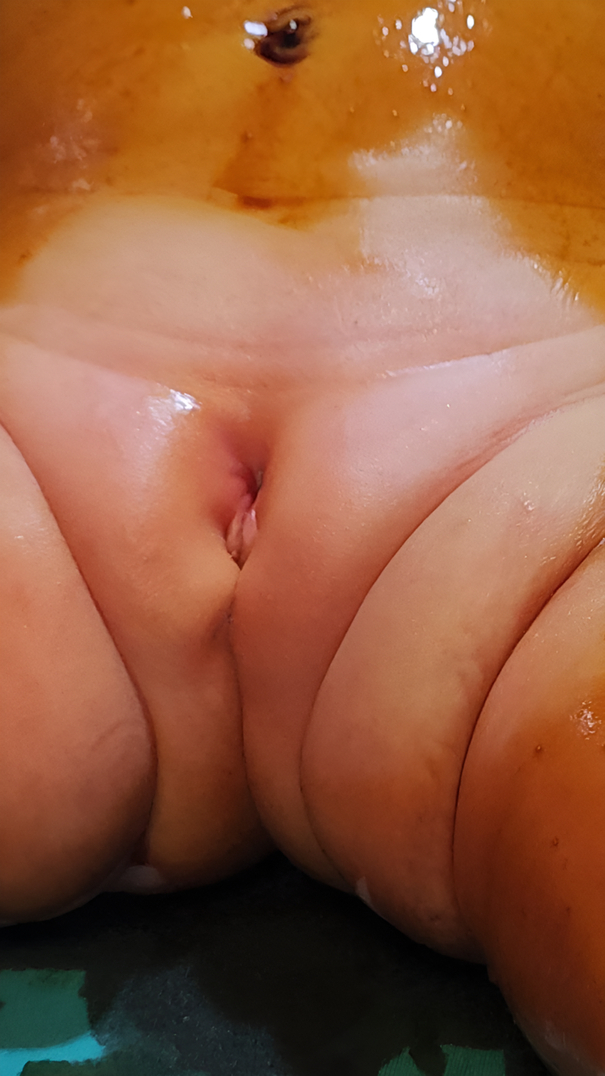
Inflammation resolution after antibiotics.

**Figure 3:**
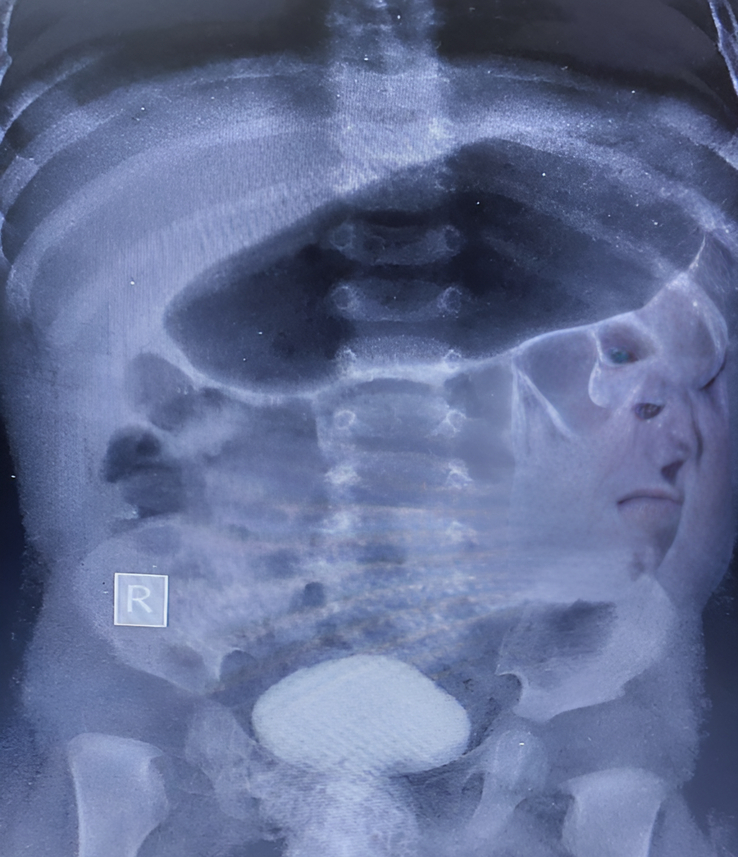
Cystourethrogram.

We suspected it to be an accessory epispadias urethra. A complete check-up evaluation was considered to support our diagnosis. Urine analysis showed bacteria in urine, so we prescribed antibiotics for the child to aid in resolving the infection and inflammation, as shown in Figure 2. Abdominopelvic ultrasound was carried out to evaluate the anatomical integrity of the liver, kidneys, ureters, bladder, ovaries, and uterus, revealing no structural abnormalities or fluid accumulations. A voiding cystourethrogram was also conducted to assess bladder function during voiding, which showed normal bladder dynamics and ureteral anatomy without associated anomalies Figure 3. Also, a cystoscopy was done under general anesthesia through a normal urethral meatus, and the normal anatomy of the urethra, bladder neck, and ureteric orifices was shown. Intravenous urography showed that the kidneys were working usually. A narrow stream of urine was observed gushing through a small opening in the prepubic region after applying pressure with a full bladder.

### Surgical procedure

One week later, we carried out the surgical procedure with general anesthesia. Before surgery, the operative site was prepared by cleaning and draping the skin to preserve a sterile field. Also, urinary drains were applied within the restriction of the normal urethral canal for the purpose of intraoperative monitoring and postoperative drainage. At the same time, another one was incorporated into the prepubic aperture to position the accessory duct. A well-defined incision was made over the prepubic region, according to the anatomical pathway of the accessory urethral tract, to provide the best visibility while reducing damage to nearby tissues. Afterwards, the surgeon carefully separated the subcutaneous tissues in order to locate and separate the accessory urethral tract. The accessory urethra was carefully distinguished from the regular urethra to prevent any harm to the functional urethra. The nearby blood vessels and nerves were meticulously preserved to avoid potential complications.

After fully exposing the accessory urethral tract, we removed the extra urethra and abnormal tissue, ensuring urinary function remained intact without affecting the nearby pubic muscles. Careful inspection was conducted after the excision to confirm that no remnants of the abnormal tract remain, which could potentially cause recurrent issues.

**Figure 4:**
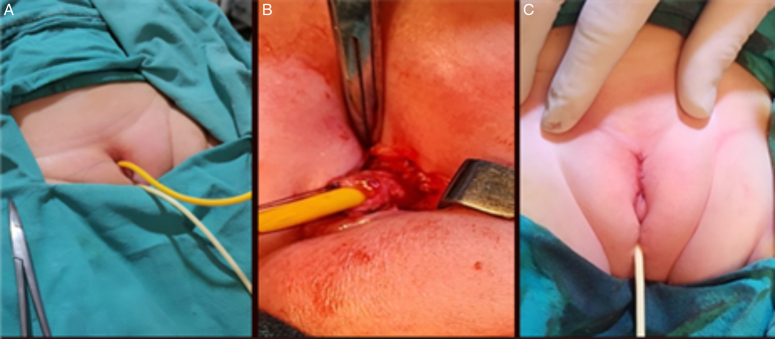
A; Foley catheter insertion in accessory and normal urethras. B: Exploring the accessory urethra. C: Final primary closure after excision of prepubic accessory urethra.

Following the excision, the wound was irrigated with saline to clear any debris or remaining blood. Hemostasis was achieved, and the closure was initiated. The wound was closed in layers, with absorbable sutures used to close deeper tissue layers, ensuring strength and minimal tension on the incision. The skin was then closed (Fig. 4).

An excisional biopsy was obtained during the procedure for histopathological evaluation, which confirmed the diagnosis of duplicated urethra and revealed mild inflammation in the urethral tissue. Postoperative care included close monitoring of urinary continence, wound management, and follow-up to ensure successful restoration of normal urinary anatomy and function without any problems. She had no complications on the day of the operation and after four days. The last follow-up after 3 months revealed a complete recovery with normal urine flow, and no other complications were found.

## Discussion

Our case represents a rare congenital condition with a low incidence, emphasizing the importance of thorough physical examinations and comprehensive diagnostic approaches, even when there is no apparent dysfunction or abnormal karyotype. Detailed clinical history, physical exams, and imaging techniques such as voiding cystourethrography, ultrasonography, MRI, or CT are crucial for diagnosing and assessing urinary tract anatomy. Surgical intervention is often required in symptomatic cases or when complications arise, with options varying based on the type of duplication. Long-term follow-up is essential to monitor for complications like strictures or symptom recurrence^5–8^.

Most of the predefined cases with urethral duplication in literature were associated with other anomalies such as double bladder, double ureter, horseshoe kidney, spinal deformities, and gastrointestinal deformities^11–14^. Our patient had a complete accessory urethra with a single bladder and did not have any other associated anomalies with normal vaginal introitus, abdominal organs, anal canal, and vertebral column.

The accessory urethra in females usually locates vaginal or sub-corporeal^15^. The accessory urethra of our patient in this study lay in a prepubic position. This position of an accessory urethra in females is rare, with most instances being accidentally identified during childhood or adulthood due to the hidden nature of the second urethral opening. Patients with complete patent urethral duplication may exhibit various symptoms ranging from asymptomatic cases to manifestations such as a split urinary stream, incontinence, urinary tract infections (UTIs), or obstructive voiding symptoms.

Accurate differentiation of true duplication often relies on radiographic imaging and intraoperative observations^6,7^.

Ismail *et al*. presented a case of a 4-year-old girl with urinary incontinence and limited urine retention. Examinations revealed a complex anomaly of the duplicated urethra with two separate openings leading to different bladder parts. The surgical intervention involved the excision of the duplicated urethra through a combined approach. The patient showed an improvement with immediate postsurgery urinary control, and the patient’s continence gradually improved^16^.

In another recent case reported by Oyinloye *et al*. a 14-year-old female with a history of urinary incontinence was diagnosed with isolated female epispadias. Despite recurrent urinary tract infections, no trauma or adverse events during pregnancy were noted. The patient underwent urogenital reconstruction using a single-stage perineal reconstruction technique, resulting in successful long-term outcomes. The patient remained dry during follow-up visits and remained fully continent even after marriage and childbirth, demonstrating the successful long-term outcomes of surgical intervention for isolated female epispadias^1^.

The most documented cases of duplicated urethra in females describe the excision process, which typically involves encircling an incision around the external opening and obliterating it. Our approach adhered to the established protocol but added a limited extravesical exposure to guarantee total excision without causing any damage to the pubic muscles.

## Conclusion

Urethral duplication in females is very rare but should be considered in girls who have had incontinence since birth. A comprehensive examination is required. Our patient’s urethral duplication is unique; it resembles a complete duplication with prepubic accessory urethra and is not associated with other abnormalities. The primary therapeutic approach is surgical management.

## Ethical approval

No IRB approval is needed, consent for publication of the case was obtained from the patient.

## Consent

Written informed consent was obtained from the patient's parents/legal guardian for publication and any accompanying images. A copy of the written consent is available for review by the Editor-in-Chief of this journal on request.

## Source of funding

This research did not receive financial support from public, commercial, or not-for-profit sectors.

## Author contribution

S.F.M. and T.A.: wrote the original manuscript; A.S.A. and T.A.: are involved in the treatment of the patient. All authors reviewed the manuscript.

## Conflicts of interest disclosure

The authors declare no conflict of interest.

## Research registration unique identifying number (UIN)

The case report does not need this registration. As well as this registry needs to pay 99 euros and we cannot pay them because we cannot afford it.

## Guarantor

Ayed Shukhyer Askar.

## Data availability statement

We did not use any datasets or uncommercial figures in our manuscript.

## Provenance and peer review

No, our paper did not receive any invitation.
